# Hydatid Cysts of the Psoas Muscle: Insights from the Past Five Years

**DOI:** 10.3390/life14101331

**Published:** 2024-10-18

**Authors:** Octavian Catalin Ciobotaru, Oana-Monica Duca, Oana Roxana Ciobotaru, Elena Stamate, Alin Ionut Piraianu, Adrian George Dumitrascu, Georgiana Bianca Constantin, Madalina Nicoleta Matei, Doina Carina Voinescu, Stefan-Alexandru Luchian

**Affiliations:** 1 Department of Clinical Surgical, Faculty of Medicine and Pharmacy, “Dunarea de Jos” University of Galati, 35, Al. I. Cuza Street, 800216 Galati, Romania; octavian.ciobotaru@ugal.ro; 2 Railway Hospital Galati, 800223 Galati, Romania; roxana.ciobotaru@ugal.ro; 3 Department of Morphological and Functional Sciences, Faculty of Medicine and Pharmacy, “Dunarea de Jos” University of Galati, 35, Al. I. Cuza Street, 800216 Galati, Romania; alin.piraianu@ugal.ro (A.I.P.); bianca.constantin@ugal.ro (G.B.C.); 4 Emergency County Hospital Braila, 810325 Braila, Romania; luchian_alexandrustefan@yahoo.com; 5 Department of Clinical Medical, Faculty of Medicine and Pharmacy, “Dunarea de Jos” University of Galati, 35, Al. I. Cuza Street, 800216 Galati, Romania; doina.voinescu@ugal.ro; 6 Emergency University Hospital of Bucharest, 050098 Bucharest, Romania; 7 Division of Hospital Internal Medicine, Department of Medicine, Mayo Clinic Florida, 4500 San Pablo Rd S, Jacksonville, FL 32224, USA; 8 Department of Dental Medicine, Faculty of Medicine and Pharmacy, “Dunarea de Jos” University of Galati, 35, Al. I. Cuza Street, 800216 Galati, Romania; madalina.matei@ugal.ro; 9 Saint John Clinical Emergency Hospital for Children, 800487 Galati, Romania; 10 Saint Apostle Andrew Emergency County Clinical Hospital, 177 Brailei St., 800578 Galati, Romania

**Keywords:** hydatid cyst, psoas muscle, echinococcosis, surgery

## Abstract

Echinococcosis represents a major public health issue and continues to be endemic in various regions around the world. Hydatid cysts are commonly located in the liver (primary site), followed by the lungs (secondary site). However, they are also found in less typical organs such as the spleen, kidneys, peritoneum, retroperitoneum, pancreas, gallbladder, and various other organs, as well as in striate muscles. Hydatid cysts involving the psoas muscle are rare, and in the past five years, only a few articles have addressed this condition due to its low incidence. Symptoms can be quite vague, and serological testing might return negative, making imaging studies crucial for an accurate diagnosis. Treatment options involve surgery alongside antiparasitic agents. Despite having a low mortality rate, the recurrence of hydatid disease remains high. This paper aims to provide new clinical data through our case presentation, while also offering a review of the cases of hydatid cysts involving the psoas muscle that have been published over the past five years.

## 1. Introduction

Hydatid cysts are caused by the parasitic tapeworm *Echinococcus*. Of the four known species of *Echinococcus*, *E. granulosus* is the most prevalent and is predominantly responsible for cystic echinococcosis [1].

The lifecycle of *Echinococcus* involves two hosts: a definitive host, typically canines such as dogs, but it can also infect foxes and hyenas; and an intermediate host, usually livestock such as pigs, sheep, and cattle. Humans can become accidental intermediate hosts. In the definitive host, the adult tapeworm releases eggs into the environment via feces, which are subsequently ingested by the intermediate host. These eggs develop into larvae, forming cysts in the liver, lungs, muscles, and various other organs of the intermediate host [2].

The liver is the most frequent site for hydatid cysts, followed by the lungs. The occurrence of cysts within muscular tissue is exceedingly rare, accounting for only 2% to 3% of cases, with psoas muscle involvement being even less frequent, reported in only 1% to 3% of cases [3,4].

Patients with cystic echinococcosis may be asymptomatic in the early stages, with clinical manifestations emerging only as the cysts enlarge [5]. Cyst rupture or the leakage of hydatid cystic material can cause an acute or chronic/intermittent allergic reaction.

The diagnosis of hydatid disease primarily relies on imaging studies and serological tests. Blood tests can detect elevated antibody levels indicative of echinococcal infection, although a negative serological result does not conclusively exclude the diagnosis. Imaging modalities such as ultrasound, computed tomography (CT), and magnetic resonance imaging (MRI) are essential for diagnosing hydatid cysts, due to their high sensitivity and specificity [6].

The treatment of hydatid cysts involves various surgical approaches—ranging from radical surgery to conservative surgery or simple tube drainage—combined with antiparasitic medications such as albendazole, administered pre- and postoperatively to mitigate the risk of recurrence [7,8].

Scolicidal agents, such as 10% formalin, 20% hypertonic saline, and 3% hydrogen peroxide, are used to sterilize the contents of hydatid cysts during surgical procedures, preventing the spread of viable parasites and reducing the risk of secondary infection or recurrence. The ideal scolicidal agent for hydatid cysts should effectively eliminate scolices without causing local or systemic toxicity; however, such an agent has yet to be developed [9].

Although hydatid cysts are well documented in the literature, the majority of published research focuses on their occurrence in common sites, such as the liver and lungs. Due to the rarity of involvement of the psoas muscle, there is a corresponding paucity of case reports addressing this atypical infection site [10].

The few available series and case reports on psoas muscle hydatid cysts often highlight the diagnostic difficulties posed by this unusual presentation. Symptoms can be nonspecific, and serological tests may provide false negative results, prompting a heightened reliance on advanced imaging techniques for the final diagnosis. Moreover, the anatomical location of the psoas muscle complicates any surgical intervention, requiring a specialized approach to effectively manage and remove the cysts while minimizing the risks and complication [11].

The scarcity of focused research on psoas muscle hydatid cysts underscores the need for further investigation that can better underline the pathophysiology, optimal diagnostic methods, and most effective treatment strategies for this rare manifestation of echinococcosis. By compiling and analyzing existing case studies, as well as contributing with new clinical data, this paper aims to address this gap in the literature and to provide a more comprehensive understanding of psoas muscle hydatid cysts.

## 2. Our Experience

**Figure 1:**
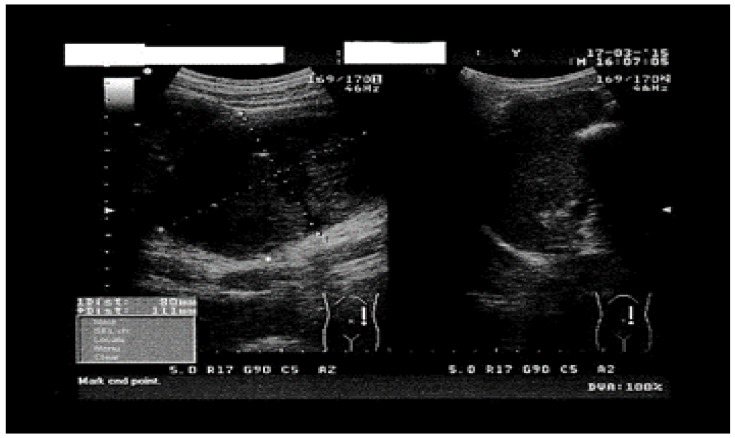
Abdominal ultrasound showing round macronodular image.

We present the case of a 41-year-old female patient, weighing 48 kg and measuring 164 cm in height (BMI = 17.91), admitted in August 2024 for treatment in the Surgical Department of Railway Hospital Galati, Romania, with dull pain in her left flank and left hypochondrium, nausea, loss of appetite, and headaches, all persistent for approximately 3 months. The patient has a cat and has not traveled outside Romania in the 5 years prior to the time of consultation. The physical examination of the abdomen revealed a mass in the left flank, poorly delimited, firm, yet elastic, adherent to the deep layers, and non-tender to palpation. Upon admission, laboratory tests showed eosinophilia (eosinophils = 0.5 × 10^3^/µL) with no other abnormalities in the complete blood count. Liver function tests and a complete renal function panel were within normal limits. Abdominal ultrasound revealed a round, solid mass located above the umbilicus, in the musculature of the posterior abdominal wall, with intra-abdominal expansion, measuring 91/75 mm in diameter. This image presented a hypoechogenic areas of 19/20 mm located intratumorally, that lacked Doppler signal, and displayed a polyseptated appearance. Additionally, another macronodular polyseptated image was identified in the left pararenal space, measuring 49/90 mm (Figure 1). The liver and spleen showed no solid lesions or cysts.

**Figure 2:**
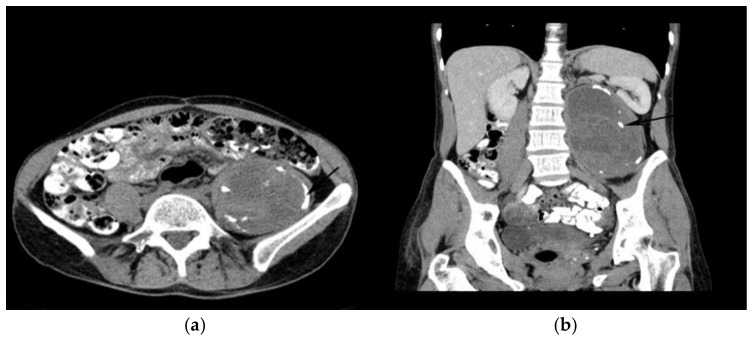
CT images in axial (**a**) and coronal (**b**) views show a distinct formation within the left psoas muscle.

To confirm the diagnosis and to assess the cyst’s relationship with surrounding structures, an abdominal CT scan with CT Siemens “SOMATOM go.UP” 32, was performed. This scan identified a well-delineated mass within the depth of the left psoas muscle, presenting a liquid-like density and parietal calcifications with impacted membranes. The cyst measured 78/86 mm in the axial plane and 130 mm in the craniocaudal plane (extending from the left renal hilum to the left iliac fossa) and causing lateral displacement of the left kidney (Figure 2a). The lesion did not enhance with intravenously administered contrast agent (Figure 2b). The image was suggestive of a hydatid cyst. No other cystic formations or structural abnormalities were identified in the liver or other abdominal organs during the CT examination. The lungs were also examined to identify any possible cysts or abnormalities, and the pulmonary radiography was normal. Serological testing for IgG antibodies against *Echinococcus granulosus* was negative. Based on the above investigations, a final diagnosis of a primary hydatid cyst in the psoas muscle was made. To treat the patient prior to surgical intervention, 12 mg/kg of oral albendazole was prescribed for 20 days.

Following this treatment, her complete blood count normalized (eosinophilia resolved), and the hepatic and renal tests remained within normal limits.

**Figure 3:**
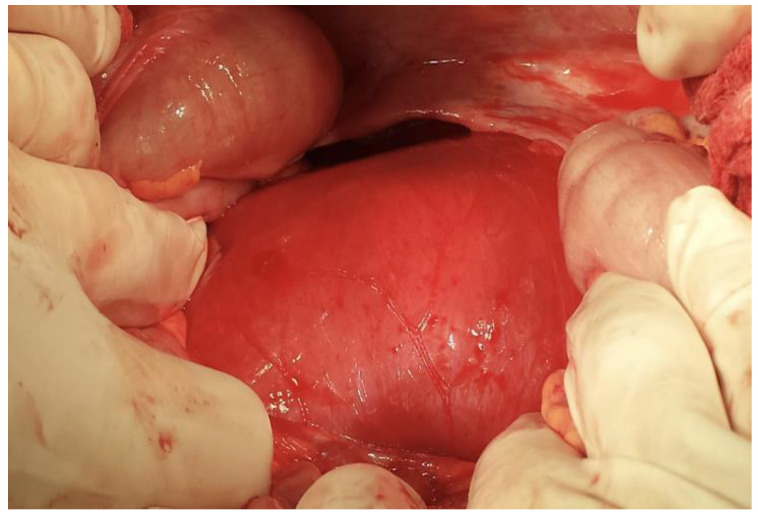
The intraoperative view reveals a tumor formation located deep within the left psoas muscle.

An exploratory laparotomy was performed. After the left colo-parietal detachment, a tumoral formation was found deep within the left psoas muscle, displacing upwards the spleen and left kidney (Figure 3).

**Figure 4:**
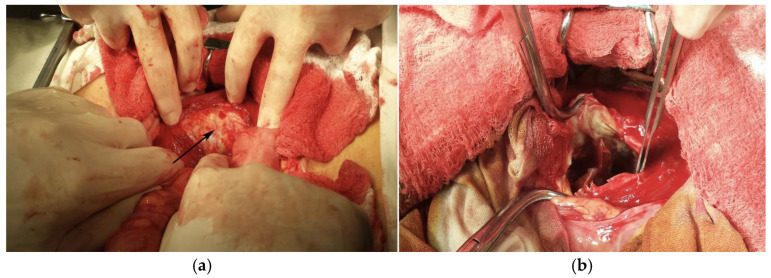
Surgical perspective showing (**a**) cystic inactivation using formalin solution and (**b**) multiple intracystic septa.

**Figure 5:**
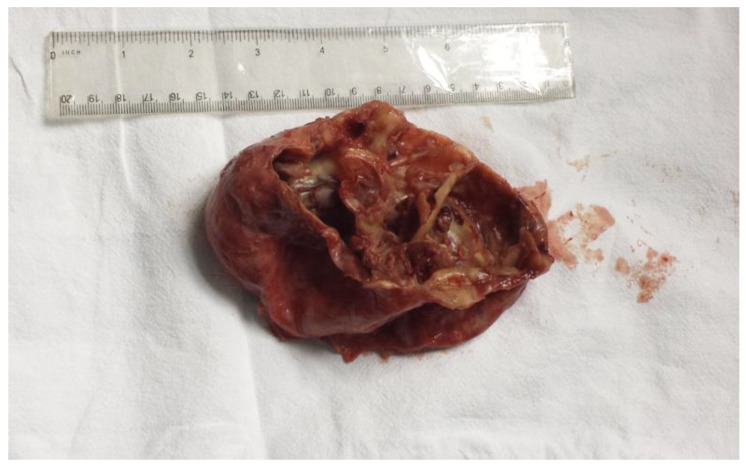
Surgical excision of two-thirds of the cyst’s perimeter.

During dissection of the left psoas muscle fibers, the hydatid cyst was exposed. The cyst was inactivated by an intracystic injection of a 10% formalin solution (Figure 4a). The operative field was isolated using betadine-stained drapes. A cystotomy was performed, discharging a chocolate-colored liquid and false membranes. Upon exploring the cystic cavity, multiple intracystic, partially calcified septa were observed (Figure 4b). A pericystic dissection was carried out to the deep strata. Due to tight relations with the vertebral bodies and the extensive fibrosis preventing further dissection, a partial cystectomy of two-thirds of the cyst’s circumference (Figure 5) was performed. The remaining cavity was cleaned with betadine-containing serum. A polyethylene drainage tube was placed in the remanent cavity and brought out through a counterincision in the left iliac fossa. Another drainage tube was placed at the bottom of the pouch of Douglas, and it was brought out through the right iliac fossa.

The surgery concluded with the layered closure of the abdominal wall and application of surgical dressing. Postoperatively, the patient received albendazole at a dosage of 10 mg/kg per day for 28 days. The anatomopathological examination confirmed the initial diagnosis of hydatid cystic formation. On the 7th postoperative day, the drain tube from the pouch of Douglas was removed. The patient’s recovery was favorable.

## 3. Material and Methods

Given the peculiarity of the case, we looked to identify within the medical literature other documented cases of psoas muscle hydatid cysts. To do so, we performed thorough searches across multiple databases, including PubMed, Google Scholar, ScienceDirect, Elsevier, Scopus, and Web of Science to locate pertinent studies and articles.

Using the search terms “hydatid cyst” and “psoas muscle,” we identified 415 published articles. We narrowed our search to include only articles published in English within the past 5 years (August 2019–August 2024) that focused exclusively on human patients.

The inclusion criteria were as follows:

- Articles published in the past 5 years, from August 2019 to August 2024.
- Articles written in English.
- Human cases of psoas muscle hydatid cysts.

The exclusion criteria were as follows:

- Articles and studies addressing hydatid cysts in locations other than the psoas muscle.
- Cases of hydatid cysts in the psoas muscle of non-human species.
- Research formats such as posters, brief papers, or abstracts only.
- Duplicate publications.
- Non-English-language articles.

After these considerations, we identified and included a total of 10 case reports, summarized in Table 1.

## 4. Discussion

The precise mechanism of how hydatid cysts develop in muscle tissue is not entirely clear. One theory posits that the cysts could form due to direct implantation, possibly from a bite or injury. Another theory proposes hematological dissemination with cysts originating from larvae that travel through the bloodstream from the intestines to muscle tissue [22].

The presence of high lactic acid concentration in skeletal muscles appears to be a significant factor that explains the rare development of hydatic cysts in these locations. The acidic environment and the contractile nature of skeletal muscles create unfavorable conditions for cyst development [23].

Clinical symptoms are often vague and may not be immediately apparent. The infection is usually found accidentally during a routine health check or during investigations for other medical concerns.

In our case, the symptoms began approximately three months prior to diagnosis. The patient initially experienced pain in the left flank and upper abdomen, accompanied by nausea, loss of appetite, and headaches.

According to the reviewed reports from the past five years, the most frequent reasons for hospital presentation in cases of psoas muscle hydatid cysts are abdominal pain, lower back pain, and discomfort in the lower limbs.

Diagnosing hydatid disease can be complex, but the combination of serological tests with imaging studies often leads to a conclusive diagnosis [24].

Serological tests play a significant role in detecting hydatid cysts but may produce false negative results, especially in cases with low antibody levels or in certain cyst locations [25].

Reviewing the selected case studies, we found that in six cases, the serological results were positive, while one case had a negative result. In the remaining four case reports, the authors did not specify the results of the serological test.

Regarding our patient, even though the serological tests were negative, the imaging results led to the suspicion of a hydatid cyst in the psoas muscle.

The use of imaging techniques such as ultrasonography, computed tomography, and magnetic resonance imaging is crucial for accurately diagnosing slow-growing cystic masses in the musculoskeletal system [26].

Based on ultrasound findings, the World Health Organizatiοn categorizes hydatic cysts into six stages [27].

The first stage involves the cystic lesion phase, where ultrasound typically shows a unilocular, anechoic cyst without internal echoes or septations.

Stages CE1 and CE2 correspond to the active phases of cystic echinococcosis (CE), where cysts are growing and are metabolically active. In contrast, stages CE3a and CE3b are transitional phases, indicating a biological shift towards the later stages of the disease, CE4 and CE5, where cysts become inactive and typically show signs of degeneration or calcification.

The images provided by abdominal ultrasound and contrast-enhanced computed tomography (CT) raised the suspicion of a hydatid cyst in the psoas muscle, despite the nonspecific symptoms and negative serological tests.

All 11 patients from the reviewed case reports were diagnosed using imaging techniques such as ultrasound, computed tomography, or magnetic resonance imaging.

The objectives of hydatid cyst treatment include parasite destruction, management of the cyst cavity, addressing potential complications, and preventing hydatid recurrences.

Albendazole (ABZ) is the preferred medication for treating cystic echinococcosis, whether used alone or in combination with percutaneous treatment.

This medication is often prescribed postoperatively to ensure the complete eradication of any remaining viable parasites, particularly in cases where cysts were manipulated or were partially resected [28].

Even if a cyst is classified as inactive (calcified or showing no signs of parasite activity on imaging or surgery), albendazole is often given as a precaution.

Inactive cysts can still harbor remnants of viable parasite material, and there is a risk of disease reactivation or secondary cyst formation if any live, infected tissue remains in place [29].

The recommended oral dosage is 10–15 mg/kg/day, divided into two doses and taken with a fat-rich meal to improve its bioavailability [30].

The usual treatment duration is 28 days, but in some cases, the course may be extended depending on risk factors, such as cyst size, location, or the occurrence of intraoperative cystic material spillage [31].

Surgical treatment for hydatid cysts is often essential for managing this condition, particularly in cases where the cysts are causing symptoms or complications [32].

Surgical therapeutic options are radical surgery (total pericystectomy, partial resection, and conservative surgery), cystotomy (surgical cyst opening to drain its contents and to manage the cystic cavity), or drainage, which involves inserting a drain to remove fluid from the cyst or its surrounding area [30].

Puncture-Aspiration-Injection-Reaspiration (PAIR) techniques are widely employed in managing cystic echinococcosis (CE), particularly during the disease’s active phases, CE1 and CE2. These techniques are minimally invasive and are conducted under ultrasound guidance to ensure precision. The procedure involves four key steps: puncturing the cyst, aspirating its contents, injecting a scolicidal agent, and then re-aspirating to remove the injected solution and any residual material [33].

PAIR is particularly effective for treating uncomplicated cysts that are not at high risk of rupture or are located in areas where surgical intervention could be more challenging [34].

In the case that we reported, the decision to proceed with surgical intervention was further supported by the progressive nature of the patient’s symptoms and the mass effect that this cyst had on the surrounding organs.

Our surgical approach involved a partial cystectomy, removing two-thirds of the cyst’s circumference. A complete resection was not possible due to the cyst’s close proximity to the vertebral bodies and the increased fibrosis, which hindered further dissection.

The main challenge of this case was the close relationship between the cyst’s capsule and the paravertebral vascular-nervous elements, as it was revealed intraoperatively. The spillage of cyst content into a vessel could have led to anaphylactic shock, and any large vessel injury during the dissection of the extremely fibrotic tissue that we encountered could have caused a significant hemorrhage, difficult to control both intraoperatively and postoperatively.

Without treatment, hydatid disease can result in complications such as infections within cysts and the formation of bacterial abscesses [35].

In the report by Moustapha T. et al., a 70-year-old patient with type II diabetes was admitted with deteriorating general health, a fever of 39 °C, and pain in the right flank. Diagnostic imaging studies conducted on admission identified a hydatid cyst in the right psoas muscle which had transformed into an abscess [14].

The disease recurrence rate is influenced by factors such as incomplete cyst removal, the spillage of cyst contents during surgery, and inadequate postoperative treatment [36].

Even though the mortality rate is low, the recurrence rate for hydatid disease remains significant. Studies indicate that recurrence can be as high as 22.2% in certain cases, underscoring the necessity for continuous post-treatment monitoring [37].

## 5. Limitations

Our review has several limitations that must be acknowledged in order to provide a balanced understanding of these findings and their implications.

First, the rarity of psoas muscle hydatid cysts inherently limits the sample size available for review. The limited number of cases studied may not fully represent the diversity of clinical presentation and the outcomes associated with this condition.

Consequently, the findings may lack generalizability, restricting their applicability to broader populations or to different geographic regions.

The retrospective nature of the reviewed studies presents another significant limitation, as previously collected data might be biased, incomplete, or inconsistently recorded.

Different researchers have used various methods for documenting or measuring data, which might lead to inconsistencies that make it difficult to compare or combine findings across studies.

Diagnostic challenges further complicate the study of psoas muscle hydatid cysts. These cysts often present with nonspecific symptoms, leading to misdiagnosis or delayed diagnosis. The reliance on imaging techniques such as ultrasound, CT, and MRI, which vary in availability and quality across different healthcare settings, introduces variability in diagnostic accuracy. This inconsistency can affect the reliability of diagnostic outcomes and the reported prevalence of the studied condition.

The variability in treatment approaches for psoas muscle hydatid cysts also poses a limitation. Different surgical techniques and the use of antiparasitic medications can vary based on regional practices and available healthcare resources. This variability can influence patient outcomes and the reported effectiveness of different therapeutic strategies, making it challenging to standardize treatment protocols and compare results across studies.

Additionally, this case report may lack comprehensive data on the long-term follow-up and outcomes of patients treated for psoas muscle hydatid cysts.

The absence of long-term follow-up data in the reviewed studies makes it difficult to assess recurrence rates and the long-term efficacy of treatment strategies.

Understanding these outcomes is essential for evaluating the overall success and sustainability of the treatments.

The exclusion of case reports published in other languages is a significant limitation of our study, as language barriers, such as translation accuracy challenges and limited resources, restricted our ability to include these cases in the review.

Lastly, the potential bias in the selection of case studies and the extent of the literature reviewed when looking for these cases are other limitations. Ensuring a diverse and representative sample of cases and studies is essential for providing a balanced and accurate portrayal of the clinical scenario.

## 6. Future Directions

Our future perspective involves broadening the research on psoas muscle hydatid cysts through several key strategies.

We aim to expand our case study collection by raising awareness among healthcare professionals, fostering collaboration, and focusing on regions where echinococcosis is endemic to build a comprehensive dataset.

Establishing detailed follow-up protocols will help track patient outcomes, including recurrence, complications, and treatment efficacy.

Collaborative efforts with research institutions and specialists are essential for advancing our understanding of hydatid cysts in rare sites like the psoas muscle, enabling knowledge exchange, standardized diagnostics, and improved research quality.

In conclusion, our future research on psoas muscle hydatid cysts will focus on expanding case studies, establishing long-term follow-up protocols, and fostering collaborative research.

## 7. Conclusions

Isolated psoas muscle hydatid cysts are rare and pose significant diagnostic and therapeutic challenges. Diagnosis is predominantly based on advanced imaging techniques such as ultrasound, CT, and MRI, as serological tests are often unreliable, particularly for cysts outside typical locations.

Standard treatment involves surgical excision, frequently combined with antiparasitic therapy, such as albendazole, to minimize the recurrence risk by sterilizing the cyst.

Although treatment outcomes are generally favorable, the recurrence rate remains high, necessitating diligent long-term follow-up.

Ongoing research and thorough case documentation are crucial for understanding this uncommon presentation and for refining treatment strategies aiming to improve long-term outcomes.

## Figures and Tables

**Table 1:** Scientific research and case presentations from the past 5 years.

Year	First Author	Country	Patient Profile	Symptoms	Serology	Imagistic	Treatment
2024	Hazem Arab [12]	Syria	40-year-old female, pulmonary hydatid cystectomy seven years previously	Abdominal pain	Not outlined	AUS CT	Cystectomy + Albendazole 15 mg/kg/d
2023	Mohammed Mhand [13]	Morocco	40-year-old male patient	Low back pain and paresthesia of the right lower limb, persisting for 2 months	Positive	CT	Partial cystectomy + Albendazole 10 mg/kg/day.
2023	Moustapha Traore [14]	Morocco	54-year-old patient	Intermittent pain and swelling in the left iliac fossa gradually increasing in size	Not outlined	CT	Partial cystectomy + Albendazole for 6 months
			70-year-old patient, history of type II diabetes	Right-sided pain developing in the context of a 39 °C fever and a decline in overall health	Not outlined	AUS CT	Pericystectomy
2022	Rajesh G. Chincholkar [15]	India	22-year-old female patient	Swelling in the right lumbar region for the previous 3 months	Not outlined	AUS CT	Cystectomy + Albendazole
2022	Atef Chamakh [16]	Tunissia	43-year-old female patient	Left lower abdominal swelling of 6 months duration	Positive	CT Pelvic MRI	Partial cystectomy + Albendazole 10 mg/kg/day for 12 weeks
2022	Dr. Youssef Bahij [17]	Morocco	28-years-old heavy smoker male patient	Lumbalgia and pain radiating towards the left thigh	Positive	CT	Pericystectomy + Albendazole 2 × 400 mg/day for 3 months
2022	Mohamed Ali Chaouch [18]	Tunisia	27-year-old male patient	Right lower abdominal quadrant pain for four months	Positive	CT	Partial cystectomy
2021	Anwar Rahali [19]	Morocco	54-year-old patient	Intermittent pain and swelling in the left iliac fossa	Negative	CT	Partial cystectomy + Albendazole 2 × 400 mg/day for 6 months
2021	Amira Atig [20]	Tunisia	70-year-old patient, diabetes and hypertension, left femoral vein thrombosis	Thrombosis of the left femoral vein	Positive	AUS CT MRI Partea superioară a formularului Partea inferioară a formularului	Surgical (no details on the technique)
2020	Yassine Merad [21]	Algeria	16-year-old female, no pathological history	Right flank pain, a few episodes of vomiting in the previous 3 days	Positive	AUS CT	Pericystectomy + Albendazole 2 × 400 mg/day for 3 months

* Abbreviations: AUS—Abdominal ultrasound; CT—Computer tomography; MRI—Magnetic resonance imaging.

## Data Availability

Data are contained within the article.
